# Sexual dimorphism in keratoconus: transcriptomic and hormonal mechanisms underlying stromal remodelling

**DOI:** 10.1186/s40662-026-00478-0

**Published:** 2026-03-03

**Authors:** Yining Sun, Qixin Li, Xiaoqing Wu, Xintong Yu, Ruoqi Wang, Binjia Sun, Kaisheng Wang, Ye Yu, Shihao Chen, Dan Jiang, Wei Chen

**Affiliations:** National Clinical Research Center for Ocular Diseases, State Key Laboratory of Ophthalmology, Optometry and Visual Science, Eye Hospital, Wenzhou Medical University, Wenzhou, 325027 China

**Keywords:** Keratoconus, Sexual dimorphism, Sex hormones, Competitive endogenous RNA

## Abstract

**Background:**

Keratoconus (KC) is a vision-threatening condition with a higher prevalence and earlier onset in males than in females. The study aims to investigate sex-associated transcriptomic features of KC and assess whether sex hormones modulate stromal remodelling.

**Methods:**

We performed whole-transcriptome sequencing of human corneal tissues from patients with KC and matched controls (n = 20; five males and five females per group), followed by weighted gene co-expression network analysis, Gene Ontology, Kyoto Encyclopaedia of Genes and Genomes enrichment, and competitive endogenous RNA (ceRNA) network construction. Our findings were functionally probed in primary human corneal stromal fibroblasts (HCSFs) using testosterone, β-oestradiol, and their antagonists. Key nodes were validated by reverse transcription-quantitative polymerase chain reaction analysis, Western blotting, and immunofluorescence.

**Results:**

Across 17,496 genes, we identified 3,345 differentially expressed genes (adjusted *P* < 0.01, |log_2_ fold-change|≥ 2). Module analyses highlighted pathways related to intracellular transport, energy metabolism, and hormone responses. Sex-stratified analyses revealed male-specific up-regulation of gonadal development programs and female-specific down-regulation of immune and hormonal processes. In HCSFs, testosterone down-regulated type I collagen but up-regulated type III collagen and α-smooth muscle actin; these effects were mitigated by flutamide. Conversely, oestrogen inhibition reproduced altered stromal remodelling, which was rapidly reversed by β-oestradiol. A female-specific ceRNA axis (circEPB41L2_0001–miR-942-5p–DCP1A) was identified and validated by performing perturbation experiments.

**Conclusions:**

KC exhibited sex-biased molecular programs consistent with androgen-driven and oestrogen-deficiency-related stromal remodelling. These findings elucidate hormone-driven mechanisms underlying KC and suggest that sex-associated hormonal regulation could inform future personalised therapeutic strategies.

**Supplementary Information:**

The online version contains supplementary material available at 10.1186/s40662-026-00478-0.

## Background

Keratoconus (KC) is a progressive and vision-threatening corneal disorder characterised by stromal thinning, biomechanical weakening, and irreversible ectasia. As the disease progresses, these pathological alterations typically lead to pronounced irregular astigmatism and can also cause stromal scarring in some advanced cases, ultimately resulting in substantial visual impairment [1]. The global pooled prevalence of KC has been estimated at 1.38 per 1000 people [1]. However, documented rates vary widely, ranging from 0.17 per 1,000 in the United States to 40 per 1,000 in Iran or even 5.3% among young Arab male students in Israel [2]. KC predominantly affects adolescents and young adults. Both genetic factors (family history) and environmental factors (including habitual eye rubbing and nocturnal ocular compression) have been implicated in KC pathogenesis [2–4]. Once considered a non-inflammatory condition, KC is now recognised as potentially involving pro-inflammatory mediators [5, 6]. Although advances in corneal collagen cross-linking and scleral contact lens use have reduced the need for corneal transplantation, KC still accounts for 4.9%–27.0% of corneal grafts across different series [7–10]. Therefore, KC continues to impose substantial socioeconomic burdens on healthcare systems [11–14].

Reports from epidemiological studies have shown a male predominance (73% of cases) and earlier disease onset in males [15–17]; however, the biological basis for these sex-associated clinical differences remains unclear. Sexual dimorphism—defined as systematic biological differences between sexes beyond reproductive traits—plays key roles in health disparities, including disease incidence, metabolic regulation, and therapeutic responses [18–20]. Sexual dimorphism also contributes to susceptibility variations in autoimmune disorders, cardiovascular diseases, and psychiatric conditions [21–23]. Despite its broad significance, the role of sexual dimorphism in KC pathogenesis has not been definitively established.

Emerging evidence points to potential sex-related mechanisms in KC. Rodríguez-Montes et al. reported that post-pubertal divergence in sex-biased gene expression coincides with the typical age of KC onset [24]. Hormonal data revealed higher salivary dehydroepiandrosterone (DHEA) sulphate levels and lower oestrone levels in patients with KC than in healthy controls [25–28]. Additionally, corneal tissues in patients with KC exhibited up-regulation of oestrogen, progesterone, and androgen receptors [29, 30]. However, research on this topic remains fragmented and lacks mechanistic validation. Importantly, whether sex differences in KC incidence and progression are driven by intrinsic biological factors (such as genetic or hormonal determinants) or external confounders (including eye-rubbing behaviours and diagnostic bias) remains unknown [31].

To address these gaps, we performed whole-transcriptome sequencing on corneal tissues from rigorously matched male and female patients with KC. This high-resolution approach enables simultaneous detection of coding RNAs, non-coding RNAs (long non-coding RNAs, circular RNAs [circRNAs], and microRNAs [miRNAs]), and splicing variants [32], providing a comprehensive view of molecular interactions that may underlie sex-related differences in KC. We observed that sex hormones were associated with KC progression, identified sex-biased genes within the cornea, and characterised a sex-specific ceRNA regulatory network. By integrating transcriptional profiles with hormonal data, our findings provide preliminary insights into the complex interplay between sex hormones and corneal homeostasis and reveal molecular pathways that may guide future hormone-modulating therapeutic strategies.

## Methods

### Patients and clinical evaluation

Participants were recruited for this research at the Affiliated Eye Hospital of Wenzhou Medical University (Supplemental Table S1). This study was conducted in accordance with the Declaration of Helsinki and was approved by our ethics committee (Reference No. 2022-068-K-50-01). Clinical data collected included demographic information (age and sex), anterior-segment photographs, corneal topography, anterior-segment optical coherence tomography, and any ocular histories. KC diagnosis was based on established criteria and global consensus [33, 34], with evaluations conducted independently by two ophthalmologists. Exclusion criteria encompassed prior ocular surgery, infectious keratitis, uveitis, or eye trauma.

### Sample collection and processing

Corneal tissues for the experimental group were excised during keratoplasty procedures performed on patients with KC at the Affiliated Eye Hospital of Wenzhou Medical University, whereas control samples were harvested from patients undergoing small incision lenticule extraction procedures for myopia correction. The control tissues consisted of intrastromal lenticules extracted from the central optical zone (6–7 mm diameter) at a depth of approximately 100–120 μm from the anterior corneal surface, with a lenticule thickness ranging from 80–120 μm depending on the degree of myopic correction. Immediately after excision, corneal tissue samples were expeditiously transferred to pre-cooled cryopreservation tubes and stored at −80 °C until RNA extraction was performed.

### RNA purification and library construction

Corneal samples from 20 participants (five male patients with KC, five female patients with KC, five male controls, and five female controls) underwent whole-transcriptome sequencing. Total RNA was extracted with the TRI Reagent (Invitrogen, Carlsbad, CA, USA) and assessed for integrity using an Agilent 2100 Bioanalyzer (Agilent Technologies, Santa Clara, CA, USA), with an RNA integrity number of ≥ 7.0 required for inclusion. Library preparation was performed using the NEBNext® Ultra™ RNA Library Prep Kit (Illumina, Inc., San Diego, CA, USA), which generates strand-specific libraries. Sequencing was performed on the Illumina NovaSeq 6000 platform (Illumina, Inc.) using 150 base pair, paired-end sequencing, with an average sequencing depth of approximately 10 Gb/sample. The raw data were converted into sequenced reads via CASAVA base calling software (v1.8; Illumina, Inc.) and stored in FASTQ format. Clean data were generated by removing adapter sequences and low-quality reads. The reference genome (hg38) and gene annotations were obtained from Ensembl (https://www.ensembl.org). Quality control analysis was performed using FastQC (v0.11.9; Babraham Institute, Cambridge, UK)**,** and reads were aligned to hg38 using Hisat2 (v2.2.1; Johns Hopkins University, Baltimore, MD, USA) [35]. Potential circRNAs were identified with find_circ [36, 37], excluding mitochondrial circRNAs. CircRNAs were retained if they were detected in at least two samples in any group (supported by at least two back-splice junction reads per sample) and had a minimum expression level of one count per million averaged across samples.

### Identification of differentially expressed circRNAs, miRNAs, and mRNAs

The DESeq2 package (v1.30.0; Bioconductor, Fred Hutchinson Cancer Center, Seattle, WA, USA) [38] in the R software (v4.3.0; R Foundation for Statistical Computing, Vienna, Austria) was used to identify differentially expressed circRNAs, miRNAs, and mRNAs. DESeq2 performs size-factor normalization and employs a negative binomial model for differential-expression analysis. Multiple testing correction was performed using the Benjamini–Hochberg false-discovery rate method. All samples were processed in a single batch to eliminate technical batch effects. Differential expression was visualised using the ggplot2 package (v3.3.5; Springer, New York, NY, USA) to generate volcano plots.

### Co-expression analysis and binding site prediction

We conducted co-expression analysis of differentially expressed circRNAs, miRNAs, and mRNAs. Pearson correlation coefficients (r <  − 0.7, *P* < 0.05) were used to identify significant circRNA–miRNA and miRNA–mRNA pairs. Potential circRNA–miRNA interactions were retrieved from circAtlas 3.0 (https://ngdc.cncb.ac.cn/circatlas) [39], which integrates Argonaute crosslinking and immunoprecipitation sequencing evidence, RNA-binding protein profiles, and multiple computational predictions to provide experimentally supported circRNA–miRNA binding relationships. miRNA–mRNA target interactions were predicted using TargetScan 8.0 (https://www.targetscan.org/vert_80/) [40], which identifies miRNA targets by integrating conserved 8mer, 7mer, and 6mer seed matches; 3′-compensatory sites; context++ scores; site conservation; and biochemical modelling-derived targeting efficacy, thereby enabling prioritization of high-confidence miRNA–mRNA interactions. Only ceRNA triplets showing inverse expression relationships consistent with ceRNA theory (i.e., circRNA–miRNA and miRNA–mRNA pairs exhibiting opposite directionality) were retained.

### Functional-enrichment analysis

The differentially expressed mRNAs underwent Kyoto Encyclopaedia of Genes and Genomes (KEGG) pathway-enrichment and Gene Ontology (GO) function-enrichment analyses using ClusterProfiler package (v3.18.0; Bioconductor) [41] and Metascape (http://metascape.org/gp/index.html). GO terms were classified into biological processes, cellular components, and molecular functions. Enrichment was considered statistically significant at *P* < 0.05.

### Weighted gene co-expression network analysis (WGCNA)

We used the WGCNA package (v1.70-3; University of California, Los Angeles, CA, USA) in R, applying a signed network approach and focusing on normalised gene expression data (log_2_-counts per million) derived from the 5,000 most variable RNAs in the KC samples. Modules were generated using the hybrid cut-tree algorithm without merging. Module eigengenes were calculated, and their correlations with KC and sex were evaluated through biweight mid-correlation analysis to identify the most strongly correlated modules for downstream analysis.

### Constructing circRNA–miRNA–mRNA ceRNA regulatory networks

A protein–protein interaction network was constructed using STRING (http://string-db.org) with a combination score threshold of > 0.4 [42]. To build the circRNA–miRNA–mRNA network, circRNA–miRNA and miRNA–mRNA interaction data were integrated and visualised using Cytoscape (v3.10.1; Cytoscape Consortium, San Diego, CA, USA) [43]. Hub genes were identified through the maximal clique centrality (MCC) algorithm implemented in the cytoHubba plugin [44]. A circRNA–miRNA–hub gene sub-network was subsequently generated to target these key regulatory genes.

### Primary culture of corneal fibroblasts

Primary HCSF cultures were established using healthy limbal tissues obtained from adult donors (15–45 years old) after corneal transplantation. The tissues were provided by the Eye Bank of Wenzhou Medical University (Supplemental Table S2). For sex-associated experiments, HCSFs from male donors were used for testosterone studies, and those from female donors were used for oestrogen studies. The epithelial and endothelial layers were removed before culturing stromal explants in Dulbecco’s modified Eagle’s medium/Nutrient Mixture F-12 (Gibco, Grand Island, NY, USA) containing 10% foetal bovine serum (Gibco). The cultures were maintained at 37 °C with 5% CO_2_, allowing sufficient time for fibroblasts migrating from the explants to reach confluency (3–5 days). The cells were dissociated with 0.05% trypsin at 37 °C for 3 min, followed by centrifugation at 300 × g for 5 min to remove the supernatant. Each cell pellet was resuspended in 5 mL of fresh culture medium, and cells were incubated in 25 cm^2^ culture flasks (Corning, Inc., Corning, NY, USA) at 37 °C with 5% CO_2_ until they reached confluence. Cells were passaged 2–3 times, and all experiments were performed using cells at uniform passage numbers.

### Cell-viability assay

To assess the dose-dependent effects of fulvestrant (MedChemExpress, Monmouth Junction, NJ, USA) and flutamide (MedChemExpress) on cell viability, HCSFs were seeded in 96-well plates at 1.0 × 10^4^ cells/well and incubated for 24 h. Then, the cells were treated with fulvestrant and flutamide at various concentrations for 12 h, washed with phosphate-buffered saline (PBS; Gibco), and incubated with 100 µL of 10% Cell Counting Kit-8 (CCK-8; Dojindo, Kumamoto, Japan) solution for 2 h. Absorbance at 450 nm was measured using a microplate reader (Molecular Devices, San Jose, CA, USA).

### Western blot analysis

HCSFs were lysed in RIPA lysis buffer (Solarbio, Beijing, China) to extract proteins. Protein concentrations were measured using a BCA Protein Assay Kit (Beyotime, Shanghai, China). Proteins were separated via sodium dodecyl sulphate–polyacrylamide gel electrophoresis and transferred to nitrocellulose membranes (GE Healthcare, Chicago, IL, USA). The membranes were blocked with 5% milk for 2 h and incubated overnight with primary antibodies at 4 °C, after which they were incubated with appropriate horseradish peroxidase-conjugated secondary antibodies for 2 h at 25 °C. Detection was performed using chemiluminescence reagents (Beyotime), and images were captured with a GE Amersham Imager AI680 (GE Healthcare Life Sciences, Chicago, IL, USA). The primary antibodies included those against type I collagen (14695-1-AP, Proteintech, Rosemont, IL, USA), type III collagen (22734-1-AP, Proteintech), and α-smooth muscle actin (SMA; 14395-1-AP, Proteintech).

### Immunofluorescence staining

HCSFs were washed three times with PBS, fixed in 4% paraformaldehyde (Solarbio) for 15 min, and rinsed with PBS three times. The cells were permeabilised with 0.5% Triton X-100 (Sigma-Aldrich) for 20 min and then washed thrice with PBS. Antibodies against type I collagen (14695-1-AP, Proteintech), type III collagen (22734-1-AP, Proteintech), and α-SMA (14395-1-AP, Proteintech) were used as primary antibodies, and anti-rabbit IgG (Alexa Fluor 594, Invitrogen) was used as a secondary antibody. Blocking was performed with 5% bovine serum albumin (BSA; Sigma-Aldrich) for 30 min, the cells were incubated overnight with primary antibodies at 4 °C in a humidified chamber, and washed thrice with PBS. Subsequently, the cells were incubated for 60 min with secondary antibodies at 25 °C in the dark. Finally, the cell nuclei were stained with 4′,6-diamidino-2-phenylindole (DAPI; Solarbio) for 5 min in the dark.

### Reverse transcription-quantitative polymerase chain reaction (RT-qPCR) analysis

Total RNA was extracted from corneal tissues using the TRI Reagent (Invitrogen) following mechanical tissue homogenisation and from cultured cells using RLT buffer (Qiagen, Hilden, Germany). DNase I treatment (Qiagen RNase-Free DNase Set; Qiagen) was performed on-column to eliminate genomic DNA contamination. The RNA was reverse-transcribed to complementary DNA using M-MLV Reverse Transcriptase (Promega, Madison, WI, USA) following the manufacturer’s instructions. The stem-loop method was employed for miRNA reverse transcription to enhance the stability and recovery of small RNAs. PCR amplification was performed using the QuantStudio™ 6 Flex System (Applied Biosystems, Foster City, CA, USA) with ChamQ Universal SYBR qPCR Master Mix (Vazyme, Nanjing, China). The 2^−ΔΔCT^ method was applied to normalise circRNA- and mRNA-expression levels to glyceraldehyde-3-phosphate dehydrogenase (GAPDH) expression and miRNA-expression levels to small nuclear RNA (U6) expression. Primers for qPCR were synthesised by Tsingke Biotechnology (Beijing, China; sequences presented in Supplemental Table S3). For circRNA quantification, divergent primers spanning the back-splice junction were used to specifically amplify circular transcripts. RNA extraction and reverse transcription protocols were optimised to retain small RNAs and minimize miRNA loss.

### RNA interference and cell transfection

We obtained two small interfering RNAs (siRNAs; siRNA1 and siRNA2) targeting the back-splice junction of circEPB41L2_0001, as well as the miR-942-5p inhibitor, from GenePharma (Shanghai, China). Cells were transfected using LipoJet™ (SignaGen, Rockville, MD, USA) following the manufacturer’s instructions, and transfection efficiency was assessed 48 h later. The relevant sequences are provided in Supplemental Table S4.

### Statistical analysis

The experimental results were expressed as the mean ± standard error and analysed using GraphPad Prism 10.1.2 (GraphPad Software Inc., La Jolla, CA, USA). Pairwise comparisons between two groups were performed using Student’s t-test. One-way analysis of variance followed by Tukey’s honestly significant difference post-hoc test was performed for comparisons of multiple groups. All experiments were performed with at least three biological replicates (n ≥ 3). Data normality was assessed using the Shapiro–Wilk test, and homogeneity of variance was verified using Levene’s test. Statistical significance was defined as *P* < 0.05. Where appropriate, effect sizes (Cohen’s d) and 95% confidence intervals are reported in figure legends.

## Results

### Differential expression of mRNAs in KC

A total of 17,496 mRNAs were commonly expressed across male and female participants in both the KC and control groups and were subjected to differential expression analysis. Overlapping expression among groups was visualised using a Venn diagram (Supplemental Fig. S1a). Principal component analysis revealed significant transcriptional differences between the KC and control groups (Supplemental Fig. S1b). Using a cut-off of |log_2_ fold-change|≥ 2 and an adjusted *P* value < 0.01, we identified 3,345 differentially expressed genes (DEGs) among the 17,496 genes, comprising 677 up-regulated and 2,668 down-regulated genes (Supplemental Fig. S1c; Supplemental Table S5). GO analysis of biological processes revealed that the up-regulated genes were mainly linked to cell–cell junctions, whereas the down-regulated genes were associated with the regulation of cell–cell adhesion and leukocyte proliferation (Supplemental Fig. S1d, e). Moreover, KEGG analysis demonstrated that the up-regulated DEGs were enriched for pathways related to glycosphingolipid biosynthesis and tight junctions, whereas the down-regulated DEGs were mainly associated with cell adhesion (Supplemental Fig. S1f, g).

### Co-expression modules identified via WGCNA

**Fig. 1:**
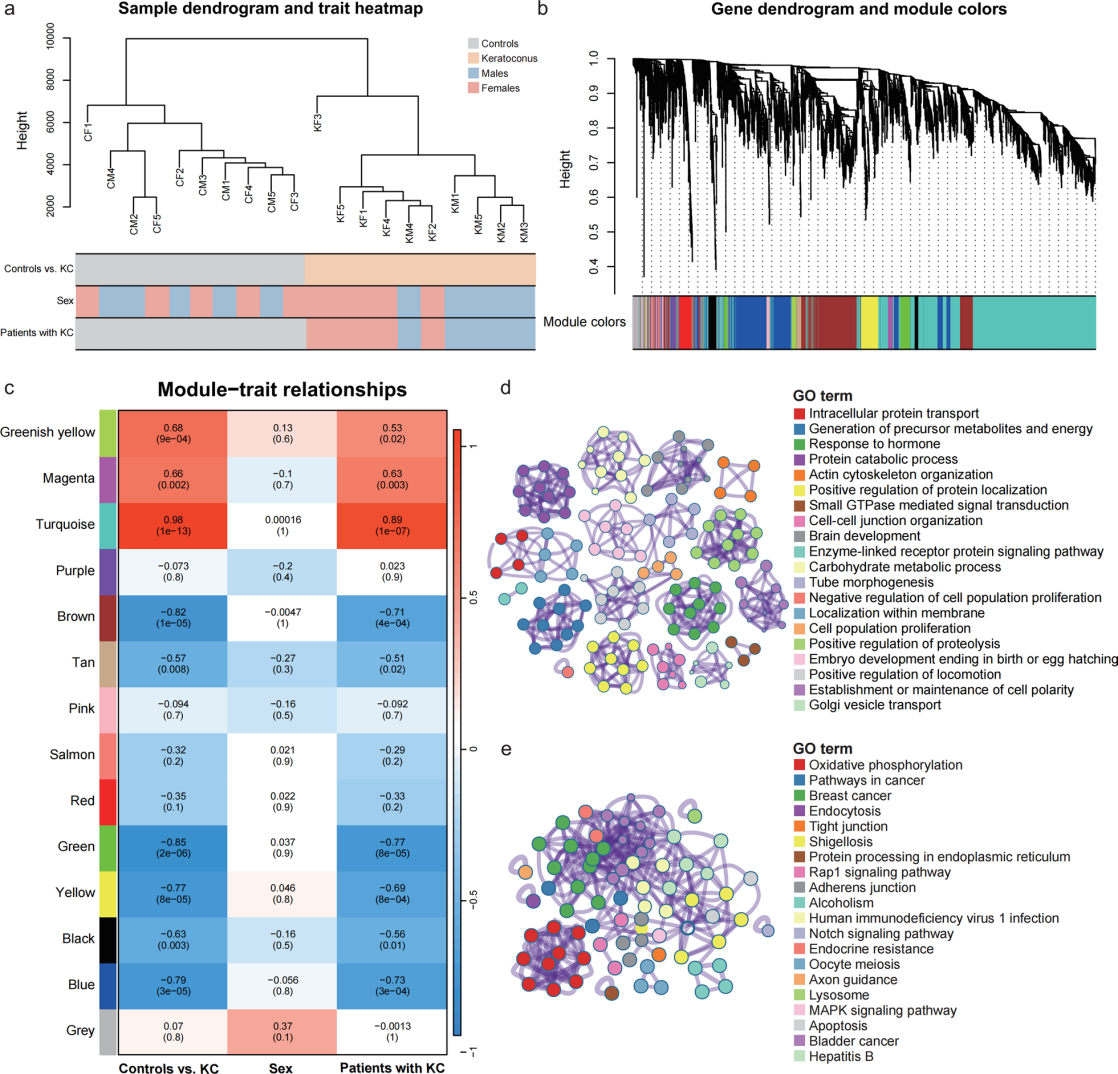
Correlations of module–trait relationships in KC. **a** Sample clustering tree. **b** Module dendrogram from WGCNA. **c** Heat map of module–trait correlations (red: positive; blue: negative); polyphenism modules are marked on the left. **d**, **e** GO enrichment analysis of genes in the turquoise module associated with (**d**) KC versus controls or (**e**) KC versus sex. CF, female control; CM, male control; GO, Gene Ontology; KC, keratoconus; KF, female with KC; KM, male with KC; WGCNA, weighted gene co-expression network analysis

Within the WGCNA framework, the pickSoftThreshold function identified an optimal soft threshold of 16, with the scale-free topology fit index (R^2^) approaching 0.9 (Supplemental Fig. S1h). Hierarchical clustering successfully distinguished the KC and control samples, forming two distinct clusters, with male and female KC patients forming separate groups (Fig. 1a). Fourteen modules were identified via co-expression analysis (Fig. 1b), with the turquoise module showing the strongest correlation with both KC and sex-associated traits (Fig. 1c). GO enrichment analysis of genes within this module revealed that KC-associated functions included intracellular protein transport, energy metabolism, and hormonal responses (Fig. 1d), whereas functions associated with sex differences included oxidative phosphorylation, endocrine resistance, and axon guidance (Fig. 1e). These results support the hypothesis that distinct pathogenic mechanisms and gene regulatory networks operate differently in male and female patients with KC, justifying independent analyses for each sex.

### Sex-specific differential-expression analysis

**Fig. 2:**
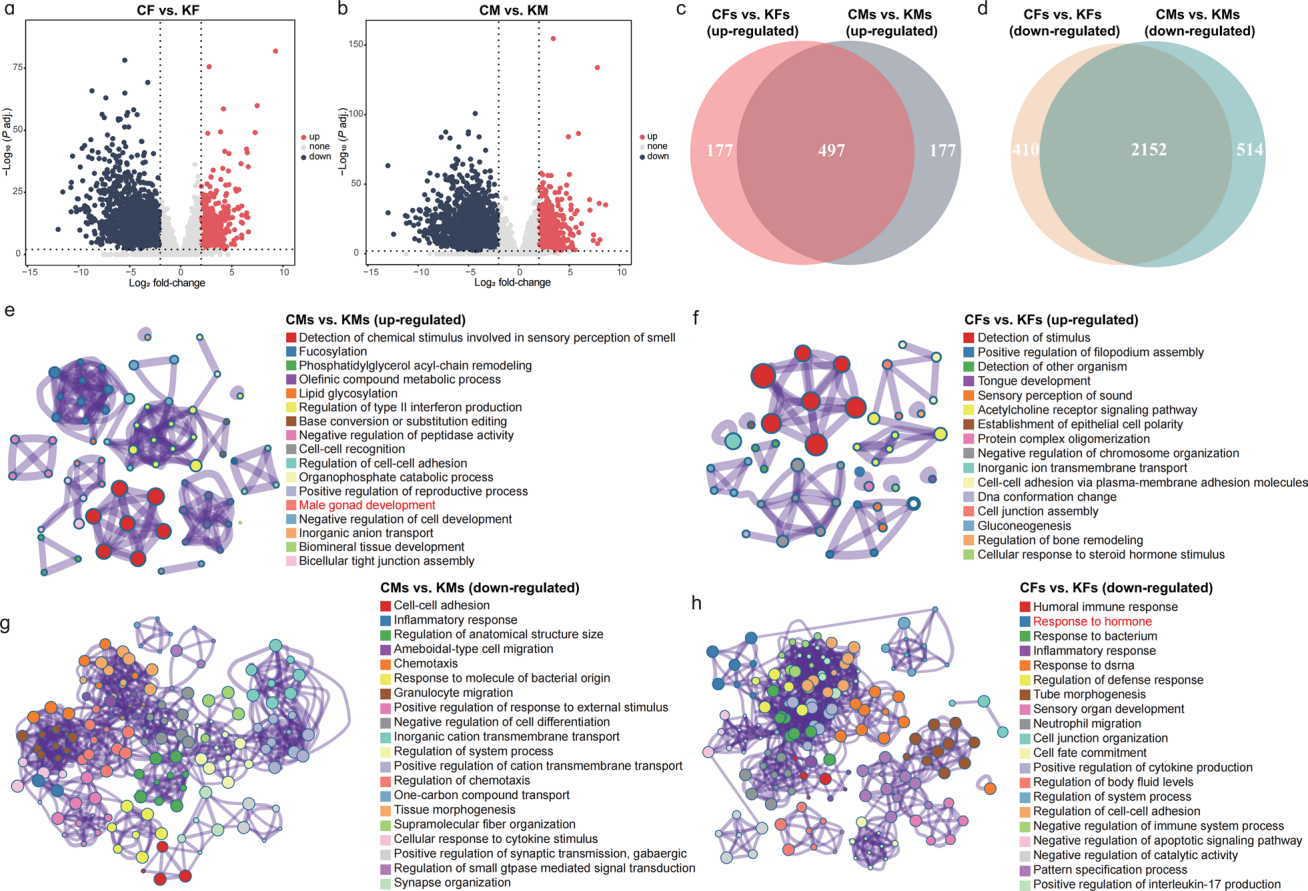
Differential gene expression in male and female patients with KC vs. controls. **a**, **b** Volcano plot differential gene expression in (**a**) KF vs. CF and (**b**) in KM vs. CM. Significance thresholds: adjusted *P* value (*P* adj.) < 0.01 and |log_2_ fold-change|≥ 2. **c**, **d** Venn diagrams showing shared and unique (**c**) up-regulated and (**d**) down-regulated genes between KM versus CM and KF versus CF. **e**, **f** GO enrichment analysis of up-regulated (**e**) male-specific and (**f**) female-specific genes. **g**, **h** GO enrichment analysis of down-regulated (**g**) male-specific and (**h**) female-specific genes. Only significantly enriched terms (false-discovery rate-adjusted *P* < 0.05) are displayed. CF, female control; CM, male control; GO, Gene Ontology; KC, keratoconus; KF, female with KC; KM, male with KC

We separately compared the corneal transcriptomes of female and male patients with KC with those of their respective controls. Using |log_2_ fold-change|≥ 2 and adjusted *P* value < 0.01 as the criteria for differential expression, we observed 674 up-regulated genes in females and 674 up-regulated genes in males, with 497 shared between both sexes. Regarding down-regulated genes, 2,666 were identified in males and 2,562 in females, with 2,152 overlapping (Fig. 2a–d). The male-specific up-regulated genes were linked to gonad development, indicating a potential role for androgens (Fig. 2e), whereas the down-regulated genes were associated with cell–cell adhesion (Fig. 2g). The female-specific up-regulated genes were related to stimulus detection (Fig. 2f), and the down-regulated genes were associated with immune responses and hormonal regulation, suggestive of impaired hormonal regulation (Fig. 2h). These findings highlight sexual dimorphism in KC pathogenesis, warranting further investigation.

### Androgen excess modulated stromal remodelling in males with KC

**Fig. 3:**
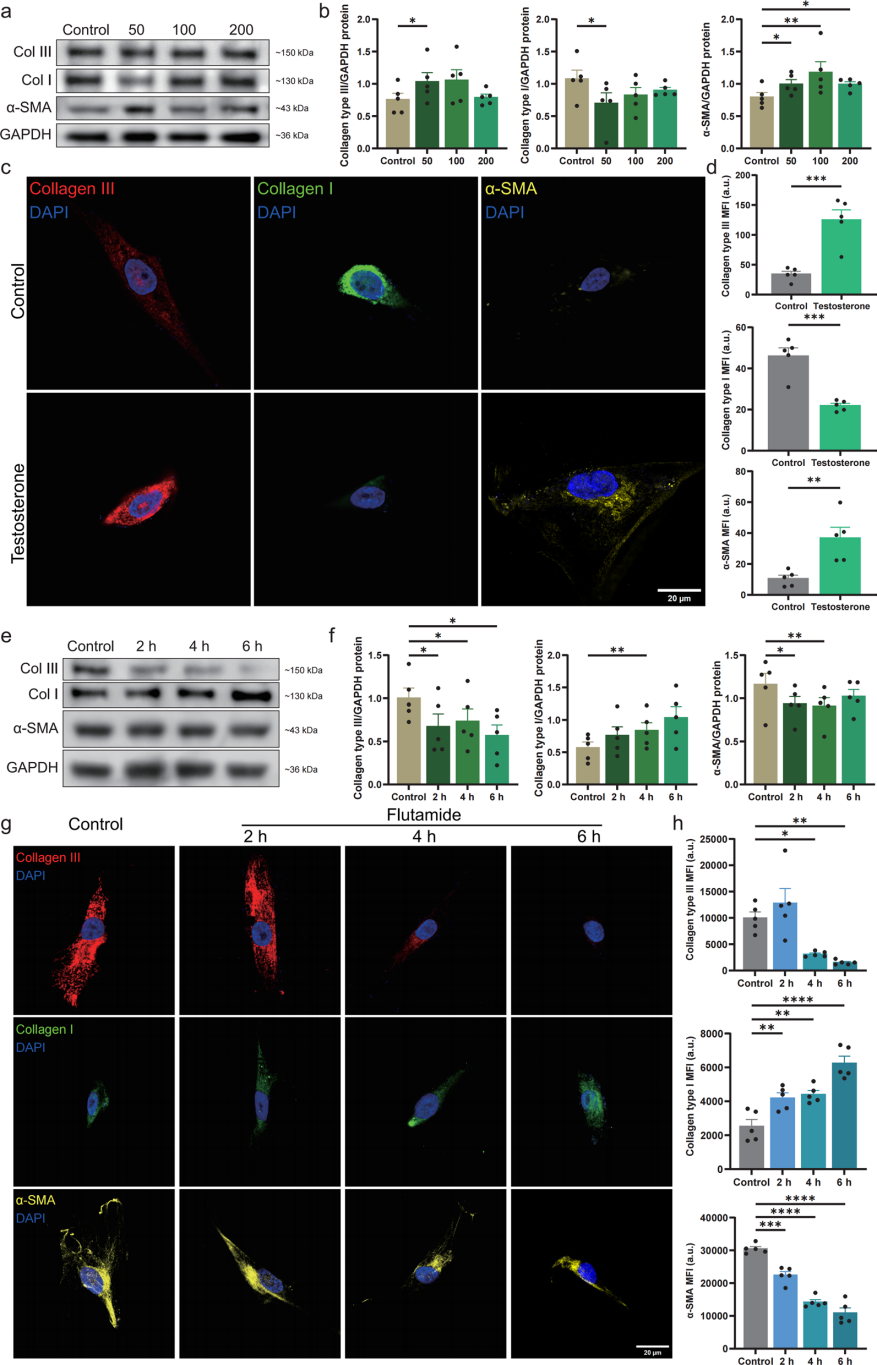
Collagen and α-SMA expression in HCSFs after testosterone stimulation. **a**, **b** Western blot showing type I/III collagen and α-SMA expression in HCSFs after testosterone stimulation (50, 100, or 200 pmol/mL; n = 5 per group). **c**, **d** Immunofluorescence staining showing protein-expression levels in HCSFs after treatment with 50 pmol/mL testosterone (n = 5 per group). **e**, **f** Western blot analysis and quantification of protein-expression levels in HCSFs after treatment with 50 pmol/mL testosterone and 10 μmol/mL flutamide (2 h, 4 h, and 6 h; n = 5 per group). Baseline expression levels in untreated cells are shown in panels (**a**) and (**b**) f(0 pmol/mL testosterone). **g**, **h** Immunofluorescence staining and quantification showing protein-expression levels in HCSFs after treatment with 50 pmol/mL testosterone and 10 μmol/mL flutamide (2 h, 4 h, and 6 h; n = 5 per group). Col, collagen; HCSF, human corneal stromal fibroblast; SMA, smooth muscle actin; GAPDH, glyceraldehyde-3-phosphate dehydrogenase; DAPI, 4′,6-diamidino-2-phenylindole; MFI, mean fluorescence intensity; *P < 0.05, **P < 0.01, ***P < 0.001, and ****P < 0.0001

To investigate the role of sex hormones in males with KC, HCSFs were treated for 12 h with testosterone at concentrations of 50, 100, and 200 pmol/mL, slightly exceeding normal levels (4.94–32.1 pmol/mL) [45]. Type III collagen and α-SMA are key stromal remodelling biomarkers, whereas type I collagen maintains corneal structure [46–48]. Western blot analysis revealed decreased type I collagen expression and increased levels of type III collagen and α-SMA, particularly at 50 pmol/mL (Fig. 3a, b). Immunofluorescence analysis confirmed these results, linking androgen excess to KC-associated stromal remodelling (Fig. 3c, d).

In evaluating reversibility, HCSFs treated with 50 pmol/mL testosterone were subsequently exposed to the androgen antagonist flutamide at an optimal dose of 10 μmol/mL, as determined using CCK-8 assays (Supplemental Fig. S2a). Flutamide treatment restored type I collagen expression and decreased α-SMA and type III collagen expression (Fig. 3e–h). These results indicate that androgen excess significantly contributed to KC in males and that inhibiting androgen activity might effectively reverse the associated stromal remodelling [28].

### Oestrogen deficiency modulated stromal remodelling in females with KC

**Fig. 4:**
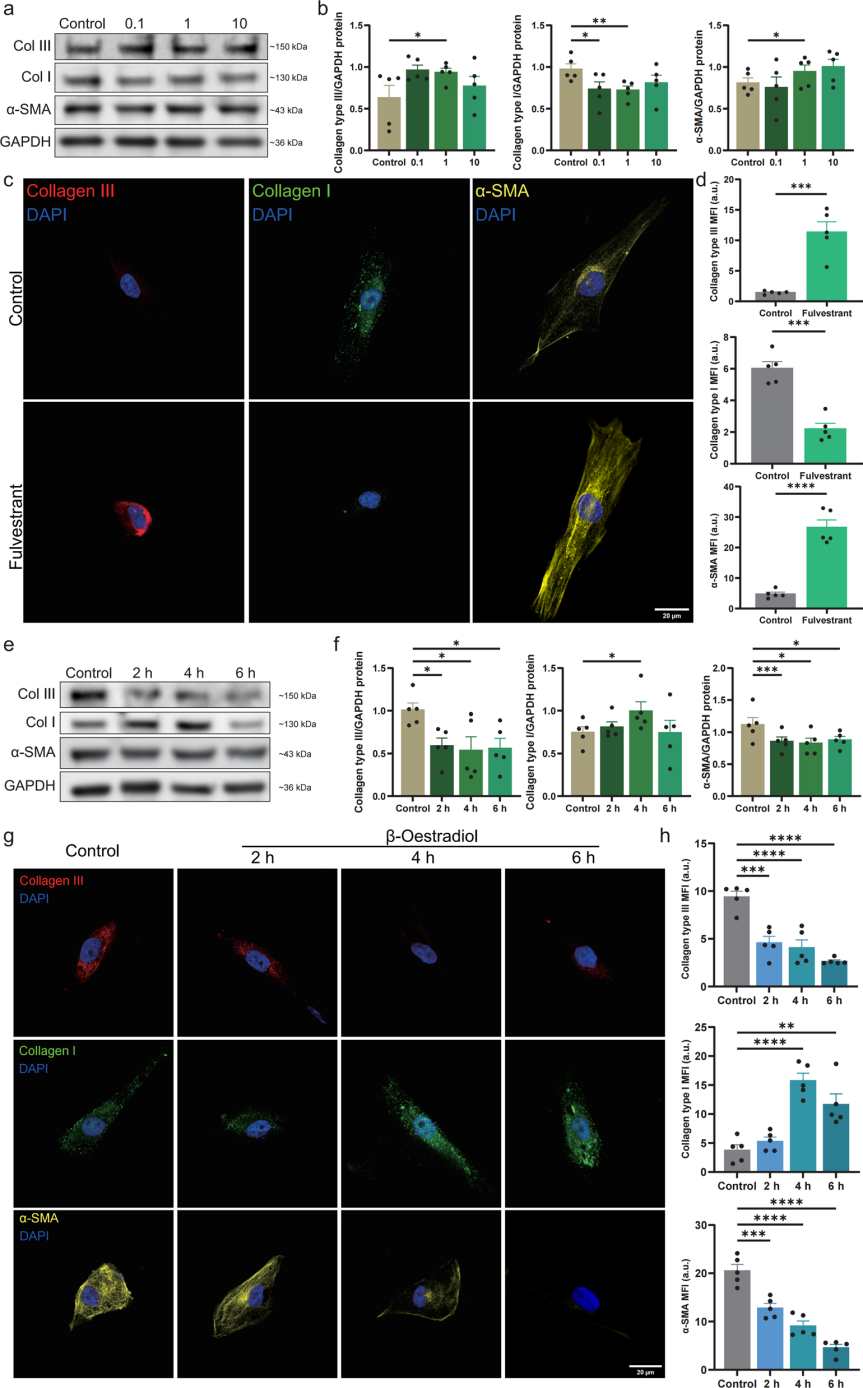
Collagen and α-SMA expression in HCSFs treated with fulvestrant and β-oestradiol. **a**, **b** Western blot analysis and quantification showing type I/III collagen and α-SMA expression in HCSFs after fulvestrant treatment (0.1, 1, and 10 μmol/mL; n = 5 per group). **c**, **d** Immunofluorescence staining and quantification showing protein-expression levels in HCSFs after 1 μmol/mL fulvestrant treatment (n = 5 per group). **e**, **f** Western blot analysis and quantification showing protein-expression levels in HCSFs after treatment with 1 μmol/mL fulvestrant and 2 pmol/mL β-oestradiol (2 h, 4 h, and 6 h; n = 5 per group). **g**, **h** Immunofluorescence staining and quantification showing protein-expression levels in HCSFs after treatment with 1 μmol/mL fulvestrant and 2 pmol/mL β-oestradiol (2 h, 4 h, and 6 h; n = 5 per group). Col, collagen; HCSF, human corneal stromal fibroblast; SMA, smooth muscle actin; GAPDH, glyceraldehyde-3-phosphate dehydrogenase; DAPI, 4′,6-diamidino-2-phenylindole; MFI, mean fluorescence intensity; *P < 0.05, **P < 0.01, ***P < 0.001, and ****P < 0.0001

We used fulvestrant, an oestrogen receptor antagonist, to pharmacologically model oestrogen deficiency in vitro. CCK-8 cell-viability assays indicated that 1 μmol/mL was the most suitable fulvestrant concentration (Supplemental Fig. S2b). HCSFs were exposed to 0.1, 1, and 10 μmol/mL fulvestrant for 12 h. Western blot analysis revealed a significant decrease in type I collagen expression following treatment with 1 μmol/mL fulvestrant in HCSFs, alongside a marked increase in both α-SMA and type III collagen levels (Fig. 4a, b). The immunofluorescence results aligned with these observations (Fig. 4c, d). These alterations paralleled the modifications seen in HCSFs from males under androgen excess conditions.

To assess reversibility, we treated HCSFs with 2 pmol/mL oestradiol after stimulating them with 1 μmol/mL fulvestrant [49]. Measurements at 2, 4, and 6 h post-oestradiol treatment showed a recovery in type I collagen expression along with reductions in α-SMA and type III collagen levels (Fig. 4e, f). Immunofluorescence analysis corroborated these observations (Fig. 4g, h). Thus, oestrogen deficiency appears to be a risk factor for altered stromal remodelling in female corneas, which could be effectively countered by administering supplemental oestrogen.

### Construction of a circRNA–miRNA–hub gene ceRNA regulatory network

**Fig. 5:**
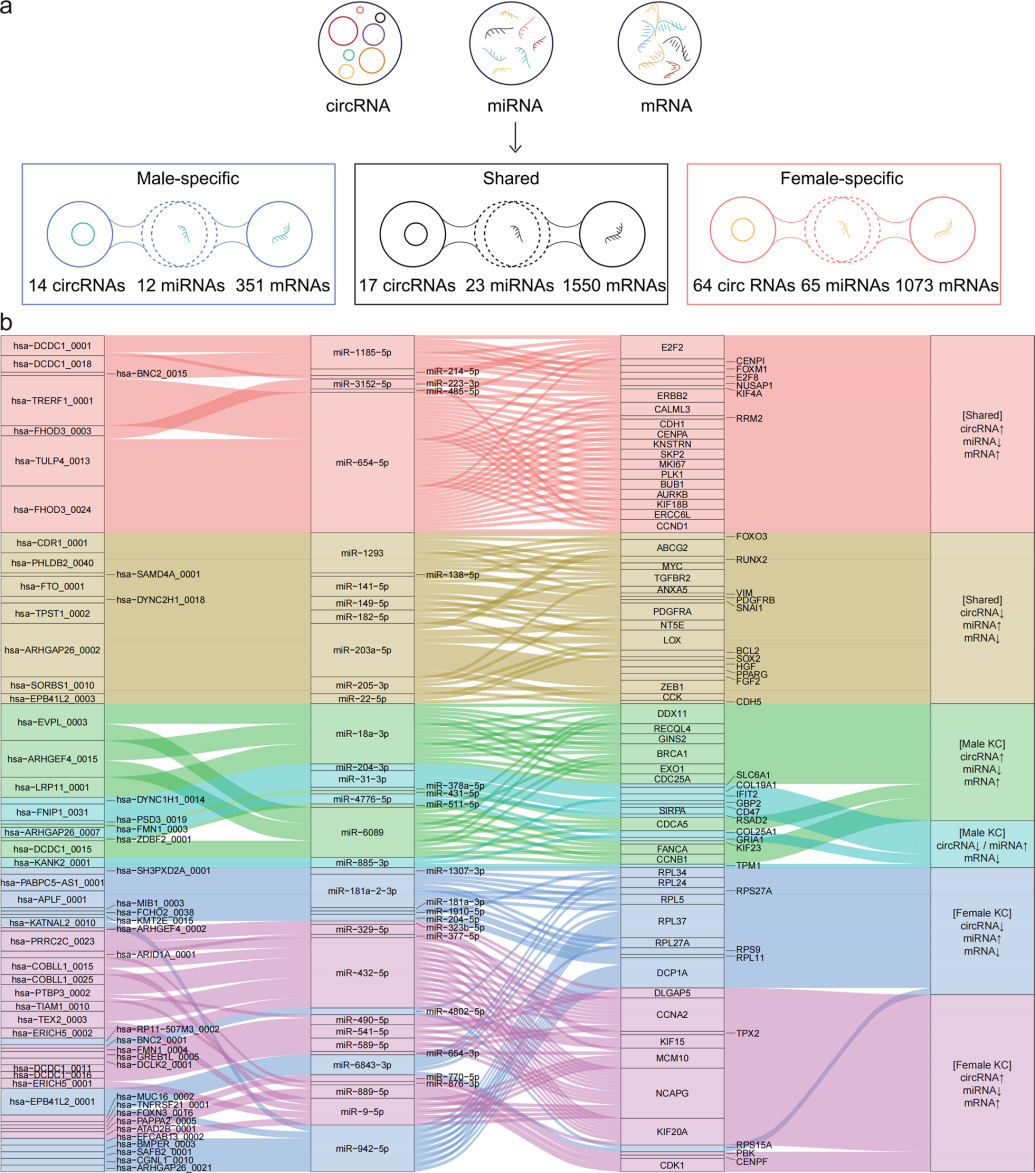
Expression profiles of circRNAs, miRNAs, and mRNAs in male and female patients with KC. **a** Diagram illustrating shared and sex-specific expression of circRNA, microRNA, and mRNA in patients with KC. **b** The circRNA–miRNA–hub gene regulatory network in KC. ceRNA interactions are illustrated based on inverse expression relationships, where circRNAs and hub mRNAs show opposite directionality relative to their corresponding miRNAs. ceRNA, competitive endogenous RNA; circRNA, circular RNA; miRNA, microRNA; mRNA, messenger RNA; KC, keratoconus

We constructed a circRNA–miRNA–mRNA ceRNA network to explore potential sex-specific regulatory mechanisms in KC. Differential expression analysis (|log_2_ fold-change|≥ 1, adjusted *P* < 0.05) identified 257 circRNAs, 322 miRNAs, and 6,523 mRNAs in males (Supplemental Tables S6–8) and 219 circRNAs, 309 miRNAs, and 6,726 mRNAs in females (Supplemental Fig. S3; Supplemental Tables S9–11). Using co-expression analysis (r <  − 0.7, *P* < 0.05) and binding-site predictions from TargetScan 8.0 and circAtlas 3.0, respectively, we identified a shared ceRNA network of 17 circRNAs, 23 miRNAs, and 1,550 mRNAs (Fig. 5a; Supplemental Table S12); a male-specific network of 14 circRNAs, 12 miRNAs, and 351 mRNAs; and a female-specific network of 64 circRNAs, 65 miRNAs, and 1,073 mRNAs (Fig. 5a; Supplemental Table S13). The MCC algorithm was used to identify 40 shared hub genes and 40 sex-specific hub genes, forming a circRNA–miRNA–hub gene regulatory network (Fig. 5b). Post-hoc power analyses confirmed high statistical power (typically > 0.95) for key ceRNA network components and hub genes highlighted in subsequent validation experiments (Supplemental Table S14).

### Validation of the circEPB41L2_0001–miR-942-5p–DCP1A ceRNA network

**Fig. 6:**
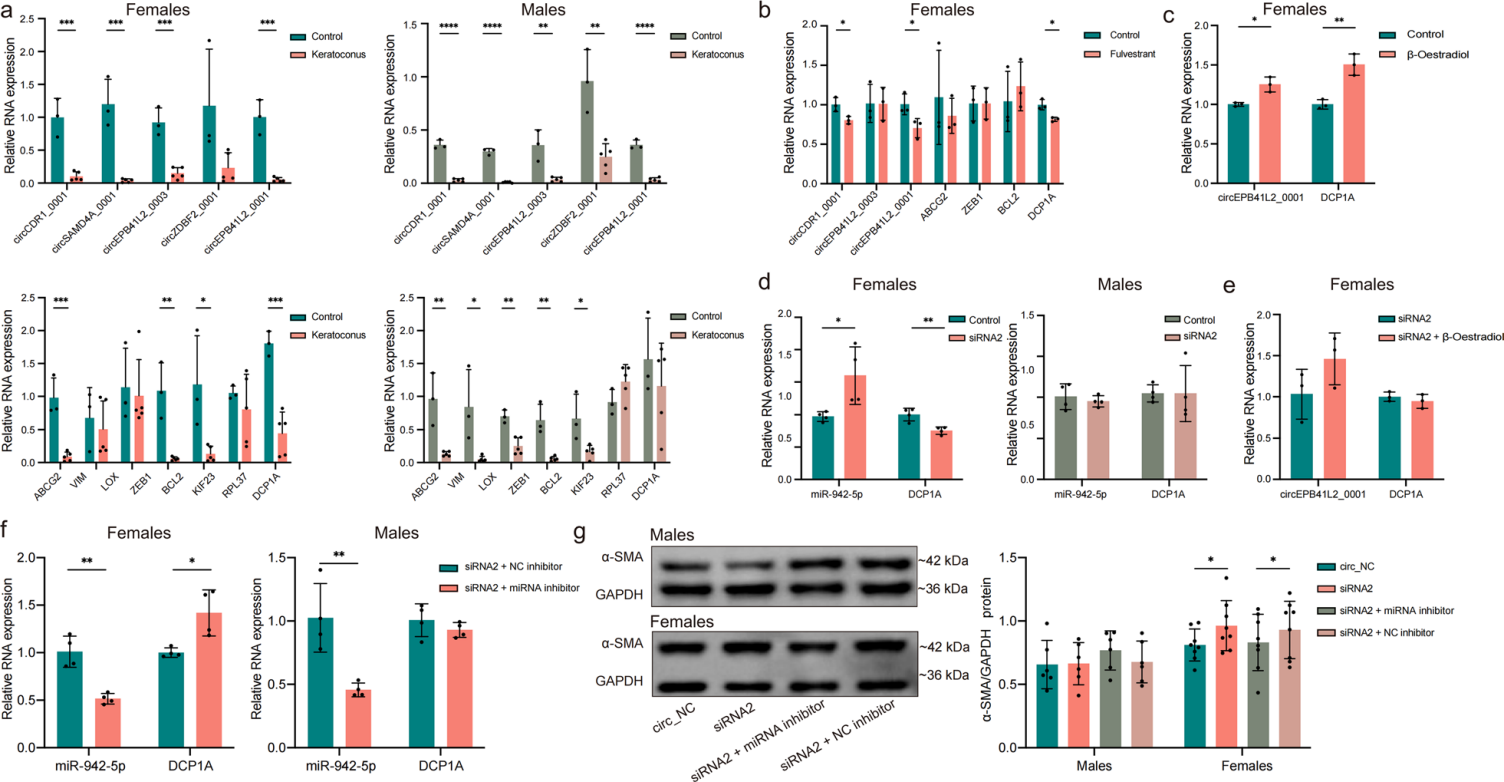
Validation of the circEPB41L2_0001–miR-942-5p–DCP1A network. **a** Relative expression levels of selected circRNAs and hub genes in normal corneal tissues vs. KC corneal tissues from male and female patients (n = 3 normal controls, n = 5 patients with KC per sex). **b** Expression levels of circRNAs and hub genes in HCSFs after fulvestrant treatment (n = 3 per group). **c** Expression levels of circEPB41L2_0001 and DCP1A in HCSFs from females following β-oestradiol stimulation (n = 3 per group). **d** Expression of miR-942-5p and DCP1A in HCSFs from males and females after treatment with siRNA2 (n = 4 per group). **e** Expression of circEPB41L2_0001 and DCP1A in HCSFs from females following combined treatment with siRNA2 and β-oestradiol (n = 3 per group). **f** Expression levels of miR-942-5p and DCP1A in HCSFs following miRNA inhibition (n = 4 per group). **g** α-SMA protein expression in HCSFs from males and females following RNA interference with siRNA2 and miRNA inhibition (n = 8 per group). *P < 0.05, **P < 0.01, ***P < 0.001, and ****P < 0.0001. Effect sizes and sex-stratified 95% confidence intervals for key comparisons are provided in Supplementary Table 15. GAPDH, glyceraldehyde-3-phosphate dehydrogenase, KC, keratoconus; miRNA, microRNA; mRNA, messenger RNA; NC, negative control; siRNA2, small-interfering RNA 2; SMA, smooth muscle actin

We validated the ceRNA networks in the corneas from patients with KC and normal controls, confirming that the circCDR1_0001, circSAMD4A_0001, circEPB41L2_0003, and circEPB41L2_0001 expression levels aligned with the RNA-sequencing data. The hub genes ABCG2, ZEB1, and BCL2 showed significant expression differences in both male and female patients, whereas VIM and LOX expression were altered only in males, and DCP1A was only altered in females (Fig. 6a). In oestrogen-deficient HCSFs treated with 1 μmol/mL fulvestrant, circEPB41L2_0001 and DCP1A were down-regulated, consistent with the changes observed in female patients (Fig. 6b). In contrast, circEPB41L2_0001 and DCP1A were up-regulated in the presence of 2 pmol/mL oestrogen (Fig. 6c). These findings suggest the existence of a female-specific ceRNA network involving circEPB41L2_0001–DCP1A.

Based on our analysis, miR-942-5p was identified as a potential component of a sex-specific ceRNA network: the circEPB41L2_0001–miR-942-5p–DCP1A network (Fig. 5b). To validate this network’s relevance to KC, siRNAs (siRNA1 and siRNA2) were designed to knock down circEPB41L2_0001 expression. siRNA2 effectively reduced circEPB41L2_0001 expression without affecting that of the parental gene EPB41L2 and was used for further experiments (Supplemental Fig. S4). circEPB41L2_0001 knockdown led to DCP1A down-regulation and miR-942-5p up-regulation in HCSFs from females, with no significant changes in HCSFs from males (Fig. 6d). When circEPB41L2_0001 expression was significantly suppressed by siRNA2, DCP1A up-regulation induced by 2 pmol/mL oestrogen was minimal (Fig. 6e), suggesting that oestrogen’s regulatory effect on DCP1A was mediated primarily through the ceRNA network rather than directly by DCP1A. Additionally, inhibiting miR-942-5p restored DCP1A expression in HCSFs from females but had no effect in HCSFs from males (Fig. 6f). Western blot analysis showed increased α-SMA expression in HCSFs from females after circEPB41L2_0001 knockdown, which was reversed by miR-942-5p inhibition, whereas no significant changes were observed in HCSFs from males (Fig. 6g). These findings confirm the existence of the female-specific ceRNA network circEPB41L2_0001–miR-942-5p–DCP1A and its association with oestrogen and KC.

## Discussion

The aetiology of KC is multifactorial and involves genetic predispositions, environmental influences, and hormonal factors [50, 51]. Recent evidence suggests that KC may involve central neuroendocrine dysregulation, including decreased circulating gonadotrophin-releasing hormone (GnRH) levels and potential involvement of the hypothalamic–pituitary–gonadal axis [52, 53]. Additionally, peripheral factors such as the body–mass index (BMI) can influence circulating sex hormone levels through adipose aromatase activity, highlighting the complex interplay between central endocrine control and peripheral metabolic regulation [54]. Through whole-transcriptome sequencing, we elucidated sexually dimorphic gene expression in KC pathogenesis, encompassing hormone-mediated HCSF responses and a novel female-specific ceRNA regulatory framework. Our findings provide valuable insights into the sex-associated mechanisms driving this disease and highlight new potential therapeutic strategies.

Our transcriptomic analysis of male patients showed that male-specific DEGs were associated with gonadal development. Notably, cross-sectional research has documented elevated serum testosterone and DHEA levels in male patients with KC [55]. Importantly, DHEA serves as the common steroidogenic precursor for both androgens and oestrogens, suggesting that hormonal dysregulation in KC may originate from upstream adrenal steroidogenesis rather than isolated downstream sex hormone alterations. Previous data indicated that males have a higher risk of developing KC, implicating androgenic factors in disease pathogenesis [15, 17]. The results of our study demonstrated that increased testosterone levels induced changes in HCSFs that were characteristic of stromal remodelling, which were reversed by flutamide treatment. Interestingly, the maximal effects on collagen metabolism were observed at 50 pmol/mL testosterone, with attenuated responses at higher concentrations, consistent with biphasic dose–response patterns commonly observed in steroid hormone signalling [56]. The association between high androgen levels and pulmonary fibrosis related to rheumatoid arthritis, as well as collagen deposition in cardiac tissues, further underscores the potential role of androgens in altering extracellular matrix compositions [57, 58]. This relationship implies that antiandrogenic therapy could exert beneficial effects by influencing extracellular matrix dynamics.

Another important finding is that fulvestrant-induced oestrogen inhibition altered stromal remodelling in HCSFs, including increased type III collagen and α-SMA levels, which were reversed by reintroducing oestrogen. This observation supports the hypothesis that menstrual cycle-associated hormonal fluctuations influence KC progression in female patients. Such fluctuations, involving variations in oestrogen and progesterone levels, can affect corneal biomechanics [53, 59, 60]. The observed hormonal effects on collagen metabolism support this hypothesis. If validated in longitudinal clinical studies, these cyclical hormonal influences could help explain the more gradual progression of KC observed in females than in males.

Our findings revealed sex-biased gene expression in KC and identified a female-specific ceRNA network: circEPB41L2_0001–miR-942-5p–DCP1A. This finding aligns with previous results indicating that circEPB41L2 can function as a molecular sponge for miR-942-5p, thereby modulating its availability and activity [61]. Additionally, elevated miR-942-5p levels have been associated with fibrosis [62]. Moreover, our findings suggest that miR-942-5p inhibited DCP1A expression. The role of DCP1A in mRNA degradation further underscores its significance, as it has been implicated in cellular proliferation and regulating cell-cycle progression [63]. These connections emphasize the multifaceted role of DCP1A in mRNA turnover and offer insights into potential molecular mechanisms warranting further investigation.

Indeed, sex-associated gene regulation is well documented in mammals and influences disease development and treatment strategies, with examples including cardiovascular disease, osteoporosis, and neurodegenerative disorders [64–66]. Such sex-biased molecular regulation extends to therapeutic responses, as demonstrated by differential microRNA-expression profiles observed in response to various treatments [67]. Collectively, our data contribute to the growing body of evidence linking noncoding RNAs and their regulatory networks to stromal remodelling, particularly within the context of sex-based differences. These findings underscore the importance of considering sex as a biological variable in research aimed at elucidating the mechanisms underlying KC and related pathologies.

Although our study provides valuable insights into hormonal influences on KC, it has a few limitations. First, our modest sample size might have reduced sensitivity for subtle sex-associated effects. Second, the absence of serum hormone measurements and BMI data, combined with the use of potentially supraphysiological hormone concentrations limits direct correlations of systemic hormones with transcriptomic changes. Future studies should include larger cohorts with emmetropic controls, comprehensive neuroendocrine profiling (GnRH and sulphated DHEA), sex hormone measurements, BMI assessments, and dose–response experiments at physiological concentration ranges to validate our findings across diverse populations. Longitudinal studies assessing hormone levels alongside clinical outcomes would provide deeper insights into the relationship between hormonal fluctuations—such as those during the menstrual cycle—and disease progression.

## Conclusions

Sexual dimorphism profoundly shapes KC pathogenesis via sex hormone-driven gene-expression changes. Elevated androgen levels in males and reduced oestrogen levels in females were associated with KC-related stromal remodelling, suggesting that hormonal regulation plays a role in disease progression. Additionally, the discovery of a female-specific circEPB41L2_0001–miR-942-5p–DCP1A ceRNA network underscores the intricate regulatory dynamics in KC. These findings provide a strong mechanistic rationale for exploring hormone-modulating strategies as potential therapies for KC.

## Supplementary Information

Additional file 1 (DOCX 581 kb)

Additional file 2 (XLSX 2705 kb)

## Data Availability

The raw RNA-sequencing data generated for this study were deposited in the Genome Sequence Archive at the National Genomics Data Center (China National Center for Bioinformation), Chinese Academy of Sciences, under accession number HRA009568. The data are publicly available at https://ngdc.cncb.ac.cn/gsa-human/browse/HRA009568. Datasets supporting the conclusions of this article are included within the article and its additional files.

## Abbreviations

BMI: Body–mass index
CCK-8: Cell Counting Kit-8
ceRNA: Competitive endogenous RNA
circRNA: Circular RNA
DEG: Differentially expressed gene
DHEA: Dehydroepiandrosterone
GnRH: Gonadotrophin-releasing hormone
GO: Gene Ontology
HCSF: Human corneal stromal fibroblast
KC: Keratoconus
KEGG: Kyoto Encyclopaedia of Genes and Genomes
MCC: Maximal clique centrality
miRNA: MicroRNA
PBS: Phosphate-buffered saline
RT-qPCR: Reverse transcription-quantitative polymerase chain reaction
siRNA: Small-interfering RNA
SMA: Smooth muscle actin
WGCNA: Weighted gene co-expression network analysis
