# Supplementary material for: Sexual dimorphism in keratoconus: transcriptomic and hormonal mechanisms underlying stromal remodelling
Source: Eye Vis (Lond). 2026 Mar 3;13:10. doi: 10.1186/s40662-026-00478-0 (PMC12955055; doi:10.1186/s40662-026-00478-0)
Supplement: Supplementary file 1 — Additional file 1 (DOCX 581 kb) [file 40662_2026_478_MOESM1_ESM.docx]

**Supplementary Materials**

**Supplemental Fig. S1.** Gene-expression profiles and pathway-enrichment analysis in KC. **a** Venn diagram of gene-expression overlaps among male and female patients with KC and controls. **b** PCA plot showing distinct clusters between patients with KC and controls. **c** Volcano plot depicting differentially expressed genes (DEGs) between the KC and control groups. Orange dots indicate up-regulated genes, blue dots indicate down-regulated genes, and grey dots represent genes that did not show significant expression differences. **d**, **e** GO-based biological process enrichment of (**d**) up-regulated and (**e**) down-regulated DEGs. The bubble size represents the number of genes enriched in each pathway, and the colour intensity indicates the adjusted *P* value (*P* adj.). **f**, **g** KEGG-based pathway enrichment of (**f**) up-regulated and (**g**) down-regulated DEGs. The bar length represents the gene count in each pathway, and the colour intensity indicates the adjusted *P* value. **h** Selection of soft-thresholding powers. FDA, false-discovery rate; GO, Gene Ontology; KC, keratoconus; KEGG, Kyoto Encyclopaedia of Genes and Genomes; PCA, principal component analysis.


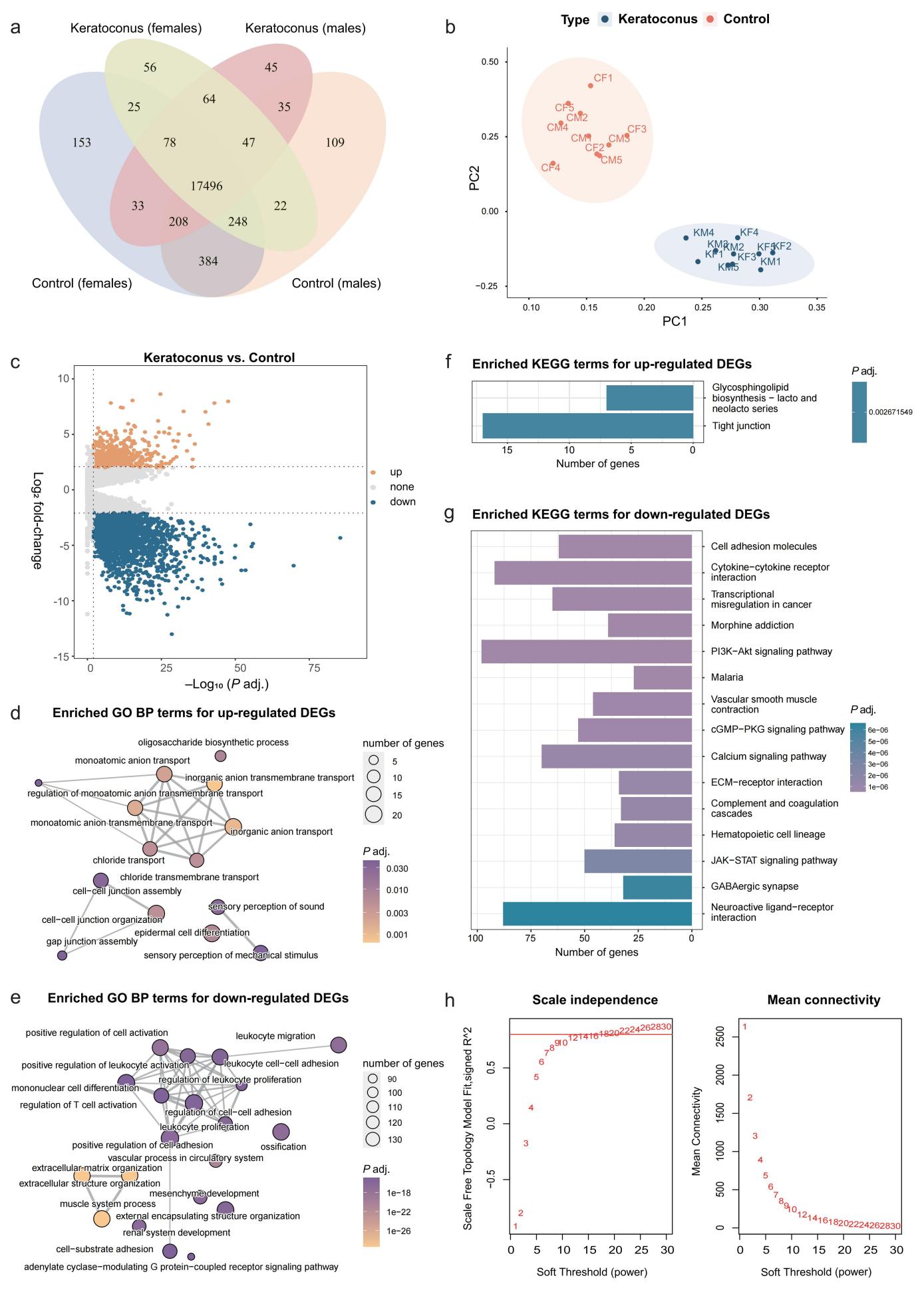


**Supplemental Fig. S2.** Impact of flutamide and fulvestrant on HCSF viability. **a** HCSF viability after exposure to increasing concentrations of flutamide. **b** HCSF viability after exposure to increasing concentrations of fulvestrant. HCSF, human corneal stromal fibroblast.

**
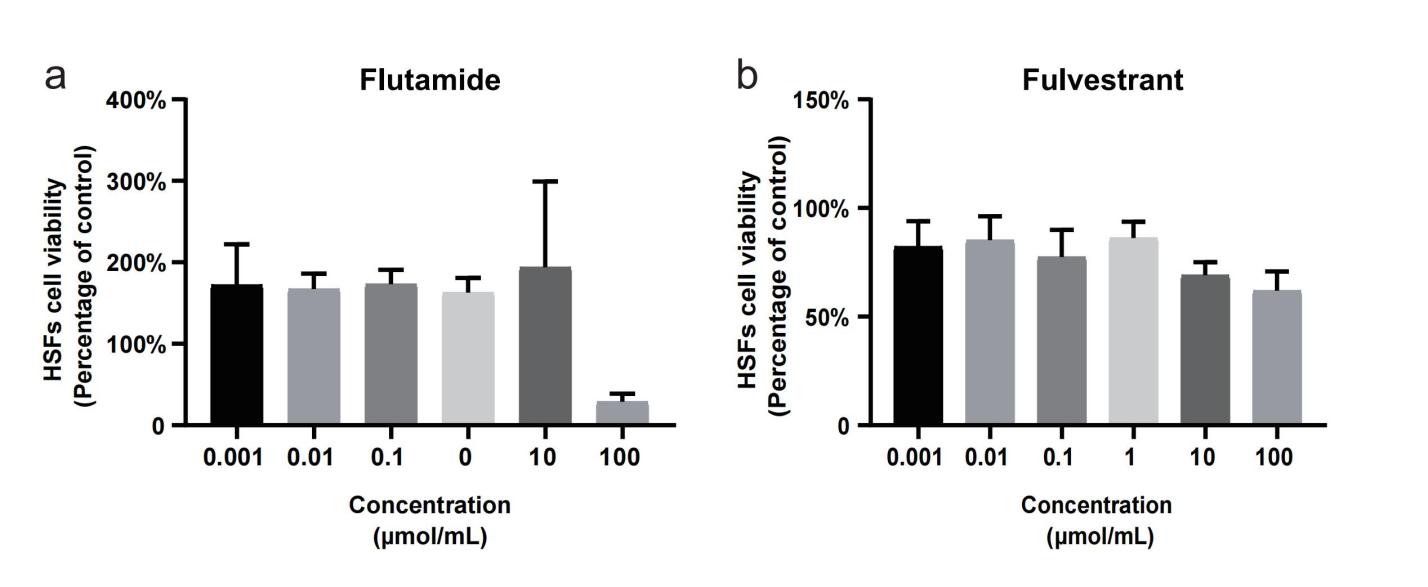
**

**Supplemental Fig. S3.** Analysis of differentially expressed circRNAs, miRNAs, and mRNAs in KC. **a–c** Differentially expressed circRNAs (**a**), miRNAs (**b**), and mRNAs (**c**) in KM vs. CM and in KF vs. CF. Red dots indicate up-regulated RNAs (adjusted *P* value < 0.05 and log_2_ fold-change > 1), blue dots indicate down-regulated RNAs (adjusted *P* value < 0.05 and log_2_ fold-change < −1), and grey dots indicate non-differentially expressed RNAs (adjusted *P* value > 0.05 or |log_2_ fold-change| < 1). circRNA, circular RNA; CF, female control; CM, male control; KC, keratoconus; KM, male with KC; KF, male with KC; miRNA, microRNA; mRNA, messenger RNA.

**
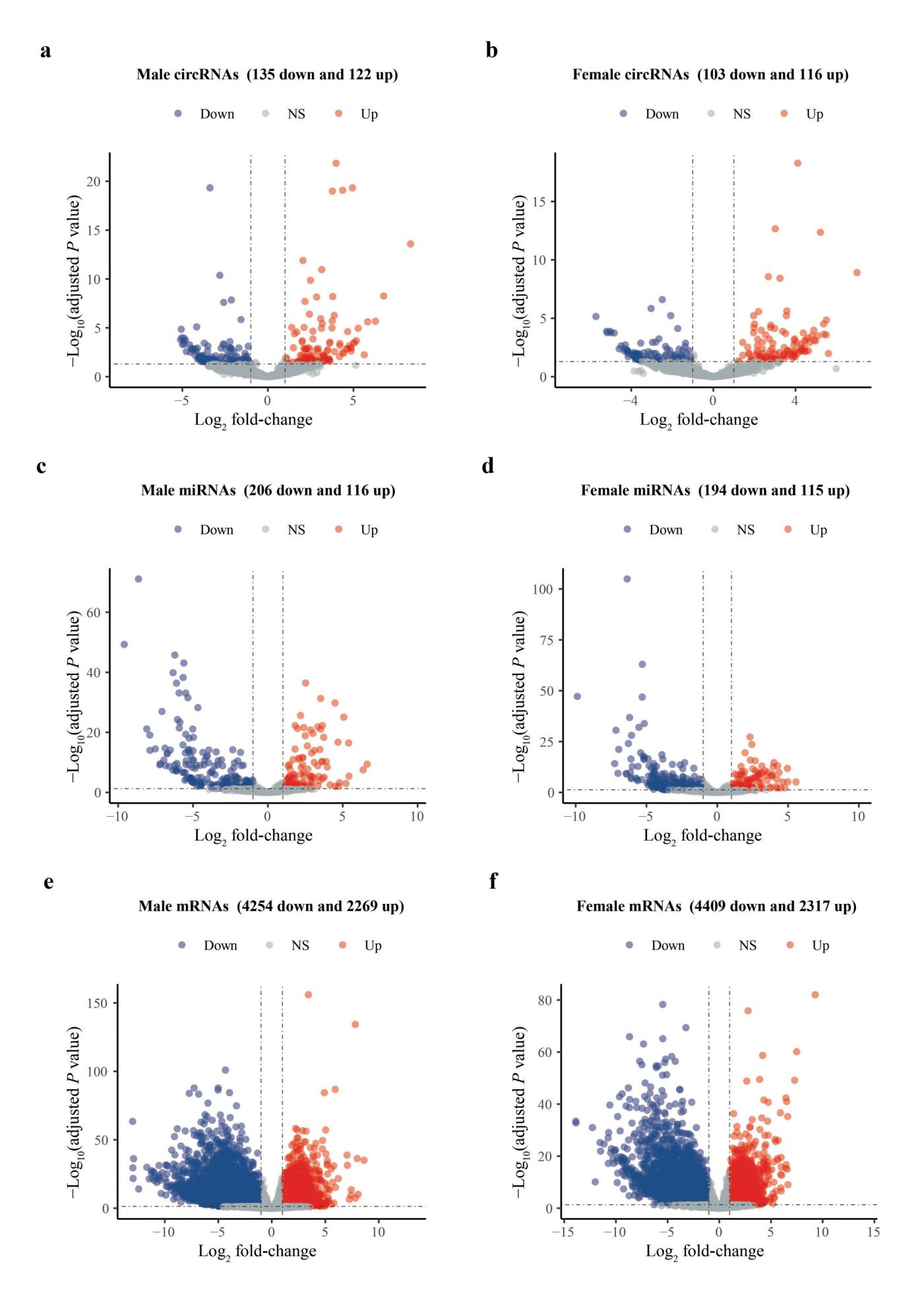
**

**Supplemental Fig. S4.** Expression of circEPB41L2_00001 and EPB41L2 in HCSFs from females and males after siRNA treatment (siRNA1 and siRNA2). circRNA, circular RNA; HCSF, human corneal stromal fibroblast; siRNA: small-interfering RNA.**
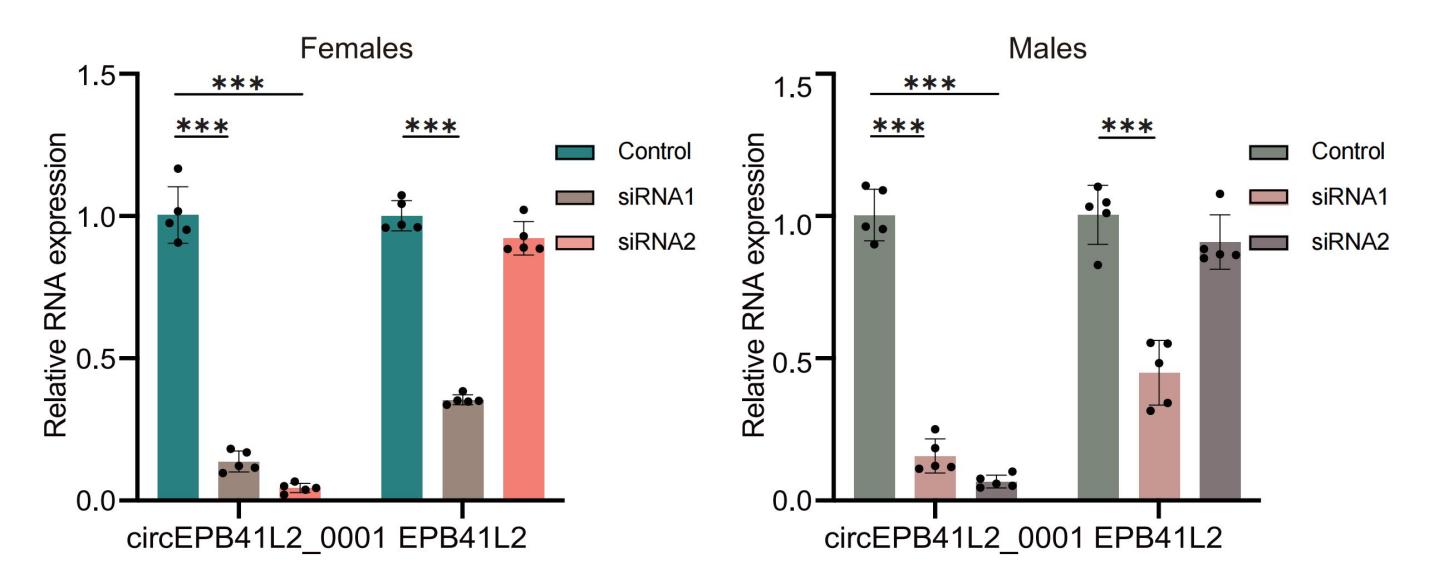
**
